# Lipoprotein Lipase Expression in Chronic Lymphocytic Leukemia: New Insights into Leukemic Progression

**DOI:** 10.3390/molecules22122083

**Published:** 2017-12-05

**Authors:** Daniel Prieto, Pablo Oppezzo

**Affiliations:** 1 Department of Developmental Neurobiology, Instituto de Investigaciones Biologicas Clemente Estable, 11400 Montevideo, Uruguay; dprieto@fcien.edu.uy; 2 Research Laboratory on Chronic Lymphocytic Leukemia, Institut Pasteur de Montevideo, 11400 Montevideo, Uruguay

**Keywords:** lipoprotein lipase, chronic lymphocytic leukemia, cancer, prognostic markers

## Abstract

Lipoprotein lipase (LPL) is a central enzyme in lipid metabolism. Due to its catalytic activity, LPL is involved in metabolic pathways exploited by various solid and hematologic malignancies to provide an extra energy source to the tumor cell. We and others described a link between the expression of LPL in the tumor cell and a poor clinical outcome of patients suffering Chronic Lymphocytic Leukemia (CLL). This leukemia is characterized by a slow accumulation of mainly quiescent clonal CD5 positive B cells that infiltrates secondary lymphoid organs, bone marrow and peripheral blood. Despite LPL being found to be a reliable molecular marker for CLL prognosis, its functional role and the molecular mechanisms regulating its expression are still matter of debate. Herein we address some of these questions reviewing the current state of the art of LPL research in CLL and providing some insights into where currently unexplored questions may lead to.

## 1. Lipoprotein Lipase

Lipoprotein lipase (LPL, EC 3.1.1.34) is a N-glycosylated protein [1] that forms homodimers and is able to hydrolize triglycerides from chylomicrons [2] and very low-density lipoproteins [3]. The first evidence of its existence was serendipitously found when studying circulating red blood cell mass in dogs. In those experiments, it was found that the administration of heparin as an anticoagulant was able to counteract alimentary lipemia in five minutes or less [4]. LPL plays a central function in lipid metabolism and has been subject of intense and meticulous studies ever since. General aspects of LPL biology have already been reviewed elsewhere [5,6].

### LPL Synthesis and Trafficking

LPL active dimer consists of two antiparallel subunits [7] whose formation and trafficking rely on a series of post-translational modifications. Interaction with calcium-dependent chaperones of the N-glycosylated polypeptide chain has been proven essential to the correct folding of LPL [8]. Furthermore, a lipase chaperon—Lipase-maturation factor 1 (Lmf1)—has been suggested to be required for dimer assembly and activity, as mutations in *LMF1* cause lipase deficiency in mice [9]. A mouse model overexpressing *LMF1* has increased LPL activity [10], and LPL has been co-immunoprecipitated with Lmf1 and Sortilin-related receptor (SorLA) [11]. It has been shown that LPL intracellular localization is regulated by SorLA, which directs its trafficking from the trans-Golgi network to endosomes [11]. LPL internalization by receptor-mediated endocytosis has been studied [12] either through LDL receptor-related protein [13] or by an LDL-receptor independent pathway [14]. 

## 2. Chronic Lymphocytic Leukemia

Chronic lymphocytic leukemia (CLL) is the most frequent form of leukemia among adult populations of Caucasian origin [15]. CLL is a malignancy of mature clonal B lymphocytes that accumulate in the blood, bone marrow and other lymphoid tissues, and is diagnosed upon the presence of ≥5000 clonal B lymphocytes per microliter of peripheral blood persisting for more than 3 months [16]. This leukemia is characterized by the accumulation of long-lived circulating clonal leukemic B-cells resulting from a complex balance between cell proliferation and apoptotic death. Increasing evidence suggests that CLL B-cells in lymph nodes (LN) and bone marrow (BM) that interact with stromal cells receive proliferative signals and are protected from cell death. These data led to the view that CLL is a dynamic process composed of cells that also proliferate and die, often at appreciable levels [17]. This crosstalk with accessory cells in specialized tissue microenvironments favors disease progression, by promoting malignant B-cell growth and the emergence of new genetic alterations which will lead to drug resistance [18]. Disease prognosis and the heterogeneous clinical evolution in CLL are probably related at least in part to these microenvironmental signaling, and although available treatments often induce remissions, CLL remains an incurable disease [19].

In CLL one third of the patients have an indolent disease with long survival and never require treatment, another third have an aggressive disease from onset and need to be immediately treated, and the last third have an indolent disease at onset which may last for years but then invariably progress to an aggressive disease [20]. It is because of this latter group that the search for strong prognostic markers in CLL predicting disease evolution has been of capital importance, and a number of them have been developed, the most reliable and universal still being the mutational status of the variable region of the heavy chain of immunoglobulin (IgHV) genes [21,22]. Patients carrying somatic hypermutation in their IgHV genes—mutated CLL (Mut)—display a better prognosis than patients with unmutated (Um) IgHV genes

## 3. LPL in Chronic Lymphocytic Leukemia

### 3.1. LPL As a Prognostic Marker of Disease Progression

Gene expression profiling analyses comparing Um and Mut patients were performed during the first decade of the century. We and others have performed these studies and described that *LPL* is differentially overexpressed in Um patients [23,24,25]. With these results in mind we selected and validated two genes, *LPL* for Um and *ADAM29* for Mut CLLs, as candidates to propose a novel prognostic method. This methodology was tested in a cohort of 127 CLL patients, and correlated to clinical outcome and IgHV mutational status. Finally, we demonstrated that quantification of *LPL* and *ADAM29* gene expression ratio is a strong prognostic indicator in CLL, providing better prognostic assessment than serologic markers in advanced stages of the disease [26]. A body of evidence has confirmed that the expression of *LPL* mRNA is associated to bad prognosis, and that it is the most robust of the molecular markers in CLL [27,28,29,30,31,32,33].

The elevated expression of *LPL* gene in Um CLL B-cells is a very remarkable observation, because there is no expression of *LPL* in normal B cells. This specific and ectopic expression constitutes not only a suitable prognostic marker to study disease evolution, but could also be helpful to understand the heterogeneous proliferative behavior in CLL. Despite the prognostic value of *LPL* expression is well established, the functional role of *LPL* overexpression in CLL pathogenesis as well as the molecular mechanisms regulating its expression are still open questions. 

Concerning the functional role of LPL in CLL cells, increasing evidence supports the idea that LPL expression could help the leukemic clone to increase survival and proliferative signaling, leading to disease progression. We have also shown that microenvironmental signaling can induce LPL expression and proliferative phenotype in primary CLL B-cells [34,35]. Supporting this idea Rozovski, Grgurevic, et al. demonstrated that LPL confers CLL a survival advantage, since shRNA knockdown of *LPL* increases apoptotic death [36]. Accordingly, it has recently been reported that *NOTCH1* gene mutations which are associated with disease progression and treatment refractoriness [37] are directly related to *LPL* expression in CLL [38]. 

Concerning the molecular mechanism that regulates *LPL* expression we previously demonstrated that abnormal expression of *LPL* gene in Um CLL patients results from the lack of methylation in the CpG island involving the whole exon 1 and the first nucleotides of intron 1 of *LPL* [34]. This epigenetic mechanism appears to be mainly triggered by proliferative T-cell-dependent signals and, in some patients, through the cross-linking of the B-cell receptor (BCR). By contrast, signaling through TLR9 or TLR1/2 pathways are unable to induce demethylation of the CpG island, *LPL* expression and B-cell proliferation [35]. Rozovski, Grgurevic, et al. have shown that *LPL* expression can also be transcriptionally regulated by STAT3 phosphorylation, and nuclear translocation where it can bind *LPL* promoter [36]. Additionally, it is necessary to mention that *LPL* expression can be regulated post-transcriptionally by miR-29 [39,40]. It has been reported that miR-29 expression is down-regulated in high-risk Um CLL patients [41,42,43,44]. In a more recent study of the microRNAome of a large patient cohort, down-regulation of miR-29c was the feature better related to IgHV Um profile [45]. In fact, Santanam et al. have developed a mouse model of early onset indolent CD5+ B-CLL by targeted overexpression of miR-29 in B-lymphocytes under control of the Eμ enhancer [46]. The authors focused on the effect on leukemogenesis by the interaction of miR-29 and *TCL1* [44,47] and did not evaluate *LPL* expression, which would be expected to be low. Deregulation of miR-29 is known to have important effects in diverse hematological disorders (reviewed in [48]), to respond to cellular signaling processes such as BCR or CD40 stimulation, and to engage NF-κB activation through TCL1 [47]. Linking these microenvironmental signaling to the epigenetic changes described by us in Um patients as well as their correlation with miR-29 and *LPL* expression could be an interesting issue that is still awaiting to be studied in CLL progression.

### 3.2. LPL in CLL B-Cell Metabolism

LPL has been shown to mediate lipolysis and subsequent fatty acid (FA)-mediated fueling of cell proliferation in several solid tumors [49], and it has recently been shown that low-density lipoproteins may enhance proliferative responses of CLL cells to inflammatory signals [50]. PPARα protein levels in CLL B-cells have been shown to correlate with leukocytosis and clinical Rai stages, which suggests a metabolic switch to oxidation of fatty acids via PPARα [51] and PPARδ [50]. These findings are supported by the observation that CLL B-cells have lipid vacuoles in their cytoplasm, and that incubation with free fatty-acids (FFAs) increased their metabolic rate in terms of oxygen consumption [36]. Furthermore, the incidence of hyperlipidemia has been found to be higher in CLL patients, and treatment of hyperlipidemia with statins benefited them in terms of a delayed time to first treatment [52]. The same group expanded their initial study to a cohort of >2000 CLL patients in a retrospective analysis and found that both lipid-lowering drugs, as well as statin treatment prolonged overall survival by 3.7 years [53]. These findings suggest that a second mechanism mediated by LDL may be converging in STAT3 phosphorylation and generating an activated state in CLL B-cells [50].

Transcriptional profiling identified a metabolic shift into a muscle or adipose tissue-like strategy with lipid oxidation in poor prognosis Um IgHV and *LPL* expressing B CLL cells [54]. How this metabolic reprogramming ends up in a worse outcome for patients is only beginning to be understood. Long chain fatty acids, free cholesterol and vitamin E- increase STAT3 phosphorylation directed either by IL-10, IFNα or phorbol esters in CLL cells [50]. STAT3 phosphorylation in turn drives *LPL* expression directly, by binding to a GAS-like element 280 bp upstream of the *LPL* transcription start site and activating its transcription [36]. LPL expression favors FA oxidation, and this seems to result in higher cell survival as *LPL* knockdown or chemical inhibition reduced CLL cell viability [36,55], which might be explained in part by a transcriptional response [32]. Accordingly, microenvironmental induction of *LPL* expression stimulates CLL cell proliferation [35]. These findings indicate that *LPL* expression can be regulated by the microenvironment, either by autocrine or paracrine signaling and that it reflects a metabolic switch in CLL B-cells which confers an adaptive advantage. A positive feedback loop may maintain *LPL* expression and worsen the scenario for Um patients. In CLL, STAT3 is constitutively activated which also activates *LPL* transcription [36]. LPL breaks down very low-density lipoproteins (VLDL) and chylomicrons and liberates FFAs, generating a proinflammatory state which in turn activates STAT3 [51] and further activation of *LPL* transcription. This would further increase the levels of FFAs, thus exacerbating CLL cells responsiveness to cytokine signaling. More general aspects of metabolic pathways in CLL have been nicely reviewed recently [56]. 

### 3.3. Non-Metabolic Roles of LPL in CLL

Many studies have reported an increased expression of *LPL* in poor prognosis CLL, and several metabolic pathways could be involved in cancer progression as discussed above. However, attempts to determine metabolic activity of LPL directly have failed to correlate higher expression to higher metabolic activity. A seminal study with 33 CLL patients reported lower catalytic activity in Um patients than in their Mut counterpart [30]. Another report analyzing data from 42 patients did not find differences between CLL groups and reported that LPL activity was comparable to that of healthy individuals [32]. 

LPL can mediate lipoprotein uptake by cells [57], chylomicron attachment to cell surface through LDL-related receptor [58], and lipoprotein margination in small blood vessels, by binding on the one hand to the extracellular surface of endothelium via GPIHBP1, and on the other to triglyceride-rich lipoproteins [59]. Besides its canonical role in lipid metabolism, an interesting—yet quite unexplored—non-metabolic function of LPL has been known for 20 years. LPL can act as a bridging molecule between cells, as in the adhesion of monocytes to endothelial cells mediated by heparan sulfate proteoglycans (HSPGs) and LPL [60], whose interaction has recently been shown to be dynamic [61]. Provided that CLL cells display HSPGs on their surface [62] and that LPL forms homodimers, it could occur that a bridging between leukemic B-cells and other cells expressing surface HSPGs or GPIHBP1 such as endothelial cells would be mediated by LPL. Although several groups have already speculated about it, a cell–cell bridging role for LPL in CLL pathogenesis still has to be demonstrated [30,35,63]. If such a bridging actually occurred, LPL would be pivoting between surface HSPGs on the B-CLL cell side, and either HSPGs or GPIHBP1 on their counterpart.

Rombout et al. have found that two SNPs commonly found in *LPL*, rs328 (premature stop codon) and rs13702 were significantly associated with CLL outcome [63]. Although both SNPs are well-known gain-of-function mutations [64,65], the authors of the aforementioned study reported not to have been able to detect significant differences in LPL mRNA, protein levels, or enzymatic activity in patients carrying the SNPs [63]. How these mutations affect clinical outcome in CLL is still unclear, but whether these SNPs might have a role—if any—in LPL non-metabolic functions has not been explored yet. Furthermore, at least nine isoelectric point isoforms of LPL have been described in human blood of healthy individuals [66], thus opening a new dimension of studies to come for LPL in CLL and other pathologies.

## 4. Concluding Remarks

**Figure 1:**
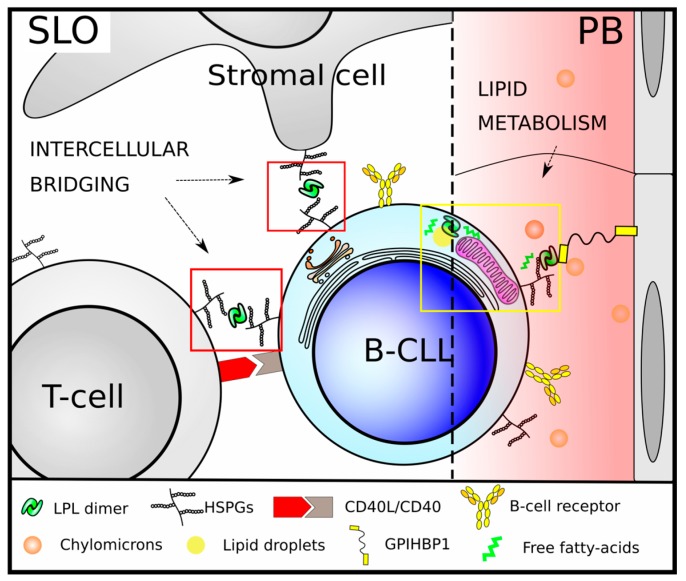
Hypothetical model of LPL function in CLL B-cells in secondary lymphoid organs (SLO, left) and peripheral blood (PB, right). HSPG-attached LPL molecules at the surface of B-CLL cells can bind very low-density lipoproteins and chylomicrons thus contributing to oxidative metabolism and fatty-acid signaling. LPL has been proposed to play a similar role in the intracellular compartment by releasing FFAs from cytosolic lipid droplets [36,56]. A non-canonical role for LPL in CLL B-cell surface would contribute to microenvironmental crosstalk. LPL would act as a bridging molecule between cells able to bind LPL either by heparan sulfate proteoglycans or GPIHBP1, thus facilitating modulatory interactions, exemplified here by a T-cell dependent activation through CD40/CD40L interaction.

LPL is a protein located on the luminal side of the blood vessel wall, where it is anchored to heparan sulfate proteoglycans and contains binding sites for both heparan sulfate chains and apoproteins [67]. *LPL* is overexpressed in B-cells of unmutated IgHV CLL patients, and its expression can be used to predict their clinical outcome [23,24,25,26,27,28,29,30,31,32,33]. Accordingly, LPL could have a bridging function in the formation of a trimolecular complex including a lipoprotein particle, LPL and heparan sulfate proteoglycans from different cells [67]. The fact that CLL B-cells display heparan sulfate proteoglycans on their surface [62], invites to speculate about whether LPL localization on the cellular membrane could affect the biological behavior of CLL cells, by favoring cell spreading, migration and intracellular signaling following activation of the tumoral clone by an activated microenvironment. If it is the case, LPL might also act as a crosstalk factor facilitating specific interactions with accessory cells in tissue microenvironments. LPL might then be added to the list of proteins implicated in the activation of CLL proliferative pool together with integrins such as CD49d, metalloproteinases (MMP-9), antiapoptotic molecules (BCL2) as well as chemokines (CCL3, CCL4, CXCL12) [68,69]. Thus, LPL could be contributing to leukemic progression either per se through metabolic reprogramming, or through the synergistic contribution to an activating microenvironment in which the leukemic clone is continuously nourished (Figure 1). 

The role that abnormal *LPL* expression could have in disease evolution, has also been addressed by previous work from Pallasch et al., demonstrating that lipase associated genes and triglyceride-specific lipase activity were significantly increased when comparing CLL B-cells to normal CD5+ B-cells [55]. The same authors reported that incubation of CLL tumoral cells with the lipase inhibitor orlistat resulted in increased apoptosis, which, could suggest that lipid metabolism and lipase activity could be functionally relevant in aggressive CLL [55]. Phenotypic analyses have shown that CLL B-cells expressing *LPL* are also enriched in FA degradation genes [54]. Recently, LPL has been shown to mediate lipolysis and subsequent FA-mediated fueling of cell proliferation in several solid tumors [49], and it has recently been shown that low-density lipoproteins may enhance proliferative responses of CLL cells to inflammatory signals [50]. 

A big amount of information is known nowadays about LPL some of which relates to CLL. Still, our understanding whether *LPL* overexpression in poor outcome CLL is a cause or consequence is poor. Many questions are still open and more answers will certainly come in next years.
